# Gender Differences in Postural Stability Among Children

**DOI:** 10.2478/v10078-012-0041-5

**Published:** 2012-07-04

**Authors:** Andrew W. Smith, Franciska F. Ulmer, Del P. Wong

**Affiliations:** 1 Department of Health and Physical Education, The Hong Kong Institute of Education, Hong Kong SAR.; 2 Department of Sport and Exercise Science, The University of Auckland, New Zealand.; 3 The Technological and Higher Education Institute of Hong Kong, Hong Kong SAR.

**Keywords:** balance, proprioception, youth, sensory weighting, centre of pressure

## Abstract

This study aimed to examine the gender differences in postural stability among 8–12 year-old children. Twenty-six children participated in this repeated measures study to measure the centre of pressure (COP) under one normal condition (CONTROL: hard surface, eyes open, and looking straight ahead) and two challenging sensory conditions (ECHB: eyes closed and head back; and EOCS: eyes open and compliant surface) in randomized order. Girls had significantly lower COP path velocity (COP-PV, p < 0.05, medium effect), smaller radial displacement (COP-RD, p < 0.05, medium effect), and lower area velocity (COP-AV, p < 0.05, medium effect) as compared to boys when the three conditions were pooled. Gender differences were found in the percentage changes in COP-RD during ECHB (p < 0.05, large effect) and EOCS (p < 0.05, medium effect), and in COP-AV during both ECHB and EOCS conditions (p < 0.05, medium effect). Postural stability performance of girls had higher correlations with age (−0.62 vs. −0.40), body mass (−0.60 vs. −0.42), foot length (−0.68 vs. −0.45), and physical activity level (−0.45 vs. 0.02), as compared to boys. Girls had better postural stability than boys but were more affected by altered sensory input information. Girls are more capable of integrating their sensory inputs, whereas boys treat each sensory input somewhat separately and rely more on somatosensory feedback. Exercises such as standing on unstable surfaces with eyes open instead of eye closed and head back are more beneficial to children’s postural stability control system.

## Introduction

Postural control is the ability to control the position of the body’s centre of mass (COM) over its base of support (BOS) to prevent the body from falling and to achieve specific functional tasks (Winter, 1995). The process by which humans maintain the integrity of their postural control is referred to as balancing (Westcott et al., 1997). Stability exists when the vertical line of gravity from the COM falls within the BOS and stability improves with a larger BOS, a lower COM, and/or a more central COM within the same BOS (Bell, 1998). Postural control is a complex process requiring integration of sensory information (somatosensory, visual and vestibular feedback) and execution of appropriate postural responses (Maurer et al., 2006). Biomechanically, the high COM of the standing human together with the correspondingly small BOS results in unstable posture as compared with quadrupedal animals. Hence, the natural consequence is spontaneous sway requiring a dynamic postural stability control system (Winter et al., 1998).

Gender differences exist in postural stability of children that vary depending on their age. Several papers have noted that girls exhibit less postural sway than boys of similar ages (Demura et al., 2006; Geldhof et al., 2006; Lee and Lin, 2007; Nolan et al., 2005; Odenrick and Sandstedt, 1984; Peterson et al., 2006; Steindl et al., 2006). Specifically, Demura and colleagues noted that boys aged 3–4 years demonstrate significantly more sway than girls even though there were no significant differences in their anthropometrics (Demura et al., 2006). Moreover, three studies focused on 9–10 year-old children (Geldhof et al., 2006; Lee and Lin, 2007; Nolan et al., 2005) also found that girls have decreased sway as compared to boys. Peterson et al. (2006) suggested that girls at the age of 7–8 years have better use of vestibular information and consequently reduce the body sway as compared to boys of the same age. In agreement with this, Odenrick and Sandstedt (1984) found that girls have better balance control than boys because sway parameters are developed earlier in girls. Generally, boys lag behind with their physical growth as well as the development of their neuromuscular system. Lebiedowska and Syczewska (2000) noted that postural stability research in children has been equivocal as to whether girls under the age of 10 years exhibit better postural stability than boys. In addition, there is no previous study examining the gender difference in postural stability under challenging sensory conditions among children.

The purpose of this study was to examine the gender differences in postural stability under normal and challenging sensory conditions in a group of healthy New Zealand children between the ages of 8 and 12 years. As shown in a previous study (Riach and Starkes, 1993), girls have earlier postural control development and exhibit more adult-like balance as compared to boys in this particular age group, we hypothesized that a) girls have better postural stability than boys in normal conditions and b) girls will maintain their stability advantage over boys under challenging sensory conditions.

## Material and Methods

The present study examined children between 8 and 12 years in a repeated measures design whereby all measurements were completed in a single visit to the Biomechanics Laboratory. Posture stability was measured under one normal condition, CONTROL (hard surface, eyes open, and looking straight ahead) and two challenging sensory conditions, ECHB (eyes closed and head back) and EOCS (eyes open and foam surface) in randomized order. In the ECHB condition, the participant’s eyes were closed removing visual information and the vestibular input was degraded by tilting the head backward 45° (Brandt et al., 1981) while in EOCS, the input from presso- and mechanoreceptors of the foot sole were altered by standing on foam surface. Thus, the suppression of one type of sensory source can be used to estimate the importance of that information to postural control and indicate how the central nervous system adapts and reorganises information provided by the remaining sensory information. The motion of the centre of pressure (COP) was measured to represent postural stability, and was compared between girls and boys.

Twenty-six children (9 girls and 17 boys) participated in this study and there was no significant difference in the demographic data between genders. The study was conducted according to the Declaration of Helsinki and the protocol was fully approved by the Human Ethics Committee before the commencement of the study. Written informed consent was received from all participants and parents after detailed explanation about the aims, benefits, and risks involved with this investigation. Participants with self-reported history of neurological or musculoskeletal conditions affecting the balance control system were excluded from the study.

Prior to testing, all participants completed a physical activity questionnaire (PAQ-C) to assess their basic activity level. Body height was measured and recorded in cm to the nearest mm. Body mass was measured to the nearest 0.1 kg with an electronic weight scale with the participant in shorts and T-shirt. BMI was calculated for each participant.

The experimental session comprised of nine balance trials, three trials each of three sensory conditions, with each trial lasting 30 seconds in order to have reliable postural sway measures (Le Clair and Riach, 1996). According to the findings of Geldhof et al. (2006) who used similar methods to the present study, the composite inter-test reliability of three trials has an ICC of 0.77. The sequence of the conditions was randomised with a one-minute rest period between conditions to avoid learning or fatigue effects.

Participants were asked to stand barefoot quietly, with each foot on a separate force platform (1Hz, Models 4060-08 and 6090, Bertec Corporation, Columbus, OH, USA) embedded in the ground. Participants used a safety harness to prevent them from injury in case of an irrecoverable balance loss. The harness has proven to be safe without impeding natural quiet standing (Freitas et al., 2005). The children stood with feet shoulder-width apart and arms hanging loosely at their sides for each trial. During the CONTROL and EOCS conditions, children were standing and gazed straight ahead at a 3 m far target. However, they were not required to fix their gaze on any particular spot. For the latter condition, a 10 cm thick layer of foam was placed on top of each force platform to interfere with somatosensory information from the feet and ankles.

The COP and torque on the force platform were calculated from the force and moment components of the force platform data. The displacement of COP is the reaction to body dynamics (Winter, 1995) and follows the neuromuscular control signal to maintain the position the COM within the BOS and achieve equilibrium (Riley et al., 1990). To obtain a quantitative description of standing ability, the following COP parameters were computed.

- COP path velocity (COP-PV): the average distance travelled by the COP per second. COP-PV is assumed to decrease with better balance performance.
- COP radial displacement (COP-RD): the mean radial distance of the COP from the centroid of the COP path over the entire trial. COP-RD data were normalized by expressing the results relative to the height of the participant. COP-RD is presumed to decrease with better balance performance.
- COP area velocity (COP-AV): the average area swept out per second of a line connecting the COP to the centroid of the COP path. COP-AV is assumed to decrease with improved balance ability (Winter, 1990).

The children completed the PAQ-C (Crocker et al., 1997), a physical activity (PA) level questionnaire designed to quantify their daily activity level, which is a guided self-administered 7-day recall measure for children. It provides a summary PA score derived from nine items, each scored on a 5-point scale. A score of 5 indicates high PA level, whereas a score of 1 indicates low PA. The PAQ-C has been suggested as one of the most reliable and valid self-administered recall instruments (Crocker et al., 1997).

Data are described as means ±SD. An independent sample t-test was used to examine the gender difference in postural stability parameters, whereas one-way ANOVA was used to examine the differences between conditions. Effect sizes (Cohen’s *d*) were calculated to determine the practical difference between girls and boys. Effect size values of 0–0.19, 0.20–0.49, 0.50–0.79 and 0.8 and above were considered to represent trivial, small, medium and large differences, respectively (Cohen, 1988). Pearson product moment correlation coefficient was used to assess the relationship between COP parameters and other variables. The magnitude of the correlations was determined using the modified scale by Hopkins (2000): trivial: r < 0.1; low: 0.1–0.3; moderate: 0.3–0.5; high: 0.5–0.7; very high: 0.7–0.9; nearly perfect > 0.9; and perfect: 1. Significance level was defined as *p* < 0.05.

## Results

Significant gender differences (*p* < 0.05) were observed in COP-PV, COP-RD and COP-AV when the three conditions were pooled (Table 1). Specifically, boys had significantly higher COPPV (*p* < 0.05, medium effect), longer COP-RD (*p* < 0.05, medium effect), and higher COP-AV (*p* < 0.05, medium effect), as compared to girls. Furthermore, COP-RD (*p* < 0.05, large effect) and COP-AV (*p* < 0.05, large effect) were significantly different between genders in CONTROL condition (Table 1), indicating the sensitivity of these two parameters in differentiating postural stability between genders in this age group.

**Figure 1:**
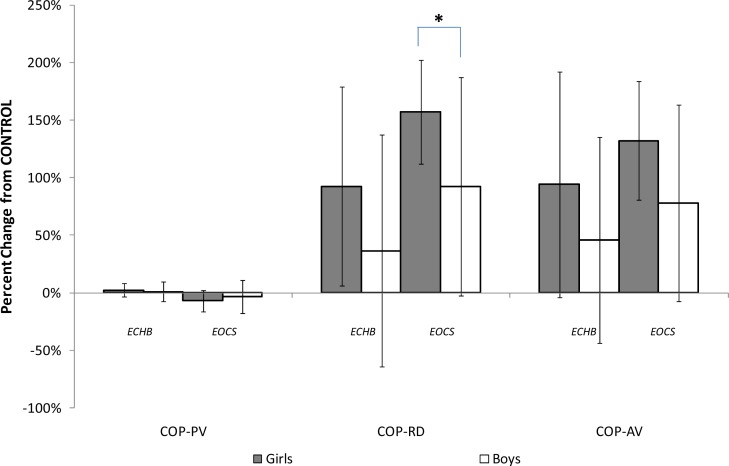
Percentage change (with reference to CONTROL) in postural stability performance for boys and girls (* indicate significant gender difference: p<0.05; COP-PV=COP path velocity; COP-RD=COP radial displacement; COP-AV=COP area velocity; ECHB=eyes closed, head tilted back at 45°; EOCS=eyes open, compliant (foam) surface)

The data in Table 1 include the analysis of the percentage change from the CONTROL condition and these data are presented in Figure 1. While there were no significant gender differences in the percentage change in COP-PV for either ECHB or EOCS, there was a significant gender difference (*p* > 0.05) in COP-RD for the ECHB condition with a medium gender effect for EOCS. There were medium gender effects in COP-AV in both ECHB and EOCS conditions.

Results of correlations showed that increases in anthropometrics, age and PA, were correlated with decreases in COP variables, i.e., improved postural stability (Table 2). Postural stability performance of girls had higher correlations with age (moderate to very high vs. moderate), body mass (moderate to very high vs. moderate to high), foot length (moderate to nearly perfect vs. moderate), and physical activity level (moderate to high vs. trivial), as compared to boys.

## Discussion

The results support our first hypothesis that girls have better postural stability than boys in normal conditions in this age group as indicated by lower path velocity, smaller radial displacement, and lower area velocity of centre of pressure (Table 2). Moreover, our results partially support the second hypothesis as there were small to medium gender differences in postural stability when performing the two challenging sensory conditions. The findings of the present study are in agreement with previous studies showing that girls in this age group have lower sway velocities (Geldhof et al., 2006) and smaller mean radius of COP distributions in both eyes-open and eyes-closed conditions (Lee and Lin, 2007) as compared to boys. In addition, several studies (Habib and Westcott, 1987; Riach and Hayes, 1987; Hirabayashi and Iwasaki, 1995) have reported that girls younger than 10 years sway less than boys. Odenrick and Sandstedt (1984) found that psychological factors (motivation and concentration) and physiological factors (differing interpretations of afferent information by the central nervous system) explain gender differences in postural stability among children at this age. In this regard, Steindl et al. (2006) found that the boys were less attentive and were more agitated during the postural stability experiment, whereas Hirabayashi and Iwasaki (1995) attributed hyperactivity as the cause of the delay in maturation of postural control in boys.

The present study provides new information to the literature by examining the percentage change from normal, quiet standing in the altered sensory input conditions (Figure 2). In this regard, boys had smaller percentage changes in postural stability performance when comparing CONTROL to the ECHB and EOCS conditions. The implications of these findings are two-fold: first, even though COP-RD and COP-AV increased in both genders (implying that the total path travelled by the COP increased), the COP-PV did not change substantially from CONTROL. Previous studies have shown the importance of controlling the COP velocity in postural stability performance (Cornilleau-Pérès et al., 2005; Jeka et al., 2004; Masani et al., 2003). Masani et al. (2003) concluded that postural control, as indicated by COM kinematics, involves a strategy relying primarily on COP velocity information and that participants used muscle activity in an anticipatory way. Jeka et al. (2004) showed that COP velocity is the most accurate form of sensory information and that changes from normal posture imposed by such manipulations as compliant surfaces and closed-eyes are mainly due to the loss of accurate COP velocity information. Cornilleau-Pérès et al. (2005) supported the use of COP velocity as a reliable and optimal indicator of postural stability in most conditions. The present study (Table 3) showed that both genders were able to control the overall velocity of their COP. However, altered sensory input information caused substantial changes in COP-RD and subsequently COP-AV that were greater in girls than in boys.

Second, the percentage change in COP-RD and COP-AV suggests that, while girls had better postural control under normal standing conditions, boys exhibited relatively less change from CONTROL when sensory inputs were removed (vision) or altered (vestibular, somatosensory). Girls showed significantly larger percentage changes in postural stability compared to boys when only somatosensory feedback provided reliable information (ECHB). Similarly, when both vision and vestibular inputs were normal and the somatosensory was altered (EOCS), the girls increased 150% (medium effect) from their normal postural stability.

The results of this study suggest that girls may be less successful than boys in shifting their sensory weighting away from the unreliable altered inputs, which resulted in the highly percentage changes in postural stability. It appears that boys treat each sensory input somewhat separately, with a bias towards somatosensory since it matures quite early (Cornilleau-Pérès et al., 2005), but with less integration than do girls. When sensory inputs become unreliable, boys exhibit smaller percentage changes from normal, quiet standing. Girls, on the other hand, seem more capable of integrating their sensory inputs, but they are still maturing and are prone to making errors in selecting appropriate sensory weightings due to the complexity of the information and their lack of experience as compared to adults. In line with this, Nolan et al. (2005) also suggested that boys probably used a different strategy from the girls in trials where their eyes were closed. The combined open- and closed-loop strategy found in the girls may develop later in boys and that boys may have different vestibular function from similarly aged girls.

These findings also challenge the contention that girls have better-developed vestibular function at this age (Peterson et al., 2006). However, caution should be used in interpreting this finding as the number of girls in the present study was relatively small. Nevertheless, the present study supports the idea of different strategies for the boys and girls, with boys possibly being more reliant on somatosensory feedback. This further suggests that during sports training, physical education lessons or any other physical activity that will ultimately improve postural stability in children (particularly in boys), it would be better, when on unstable surfaces, that children train with both eyes open and avoid conditions where the eyes are closed and the head is tilted back.

The acceleration of the body depends on the total mass of the body, which is large enough to ensure that the rapid and small adjustments of muscle activity during quiet stance produce faster changes of the COP than the body itself (Day et al., 1993). Therefore, the extent to which one controls the COP in standing is clearly indicative of the quality of postural stability. In the present study, the girls were heavier (medium effect) and taller (medium effect) than the boys (Table 1). Lee and Lin (2007) have suggested that differences attributed to gender in children may be due to other factors related more to body somatotype that is independent of body size alone. They further attributed the gender differences that they found in children to the factor that the boys had larger body mass and moments of inertia. Geldhof et al. (2006) felt their gender difference data supported the conclusions of Nolan et al. (2005) and agreed that gender differences were not dependent on anthropometrics.

To maintain postural stability, the muscular forces shift the COP and this accelerates the body in the opposite direction (Day et al., 1993). The rate of movement of the COP depends upon the buildup of muscular force. In the present study, there were no correlations between postural stability measures and PA level for the boys even though the significant correlations for the girls indicated that higher PA scores were correlated with better postural stability values. This suggests that PA might play a relatively important role in the development of muscular force and subsequently, postural stability in girls.

## Figures and Tables

**Table 1:** Gender difference in postural stability performance and percentage change from CONTROL in postural stability performance for girls and boys with effect sizes, effect size magnitudes and 95% confidence intervals

	**Girl (n = 9)**	**Boy (n = 17)**	**Effect size**	**Magnitude (95% CI)**
**COP-PV (mm/s)**				
*Pooled*	8.93 ± 3.38^*^	10.86 ± 2.86^*^	0.63	Medium (−1.44/0.21)
*CONTROL*	9.11 ± 3.61	11.04 ± 3.24	0.57	Medium (−1.38/0.27)
*ECHB*	9.33 ± 3.76	11.00 ± 2.78	0.53	Medium (−1.33/0.31)
*EOCS*	8.35 ± 3.06	10.54 ± 2.69	0.78	Medium (−1.59/0.08)
**COP-PV Percentage Change from *CONTROL***				
*ECHB*	2.45 ± 5.9	0.95 ± 9.04	0.18	Trivial (−0.63/0.99)
*EOCS*	−6.88 ± 8.43	−3.16 ± 14.47	0.29	Small (−1.09/0.53)
**COP-RD (mm/m)**				
*Pooled*	5.62 ± 2.40^*^	7.04 ± 2.83^*^	0.53	Medium (−1.33/0.31)
*CONTROL*	4.06 ± 1.61^*^	6.75 ± 2.93^*^	1.05	Large (−1.87/−0.16)
*ECHB*	7.20 ± 2.20	8.79 ± 3.73	0.48	Small (−1.28/0.35)
*EOCS*	9.29 ± 1.98	11.13 ± 3.21	0.64	Medium (−1.45/0.20)
**COP-RD Percentage Change from *CONTROL***				
*ECHB*	92.44 ± 86.42^*^	36.56 ± 45.09^*^	0.90	Large (0.03/1.71)
*EOCS*	157.14 ± 100.52	92.32 ± 94.85	0.67	Medium (−0.18/1.48)
**COP-AV (mm^2^/s)**				
*Pooled*	21.57 ± 14.08^*^	33.32 ± 18.00^*^	0.70	Medium (−1.51/0.15)
*CONTROL*	13.27 ± 8.33^*^	25.97 ± 16.05^*^	0.91	Large (−1.72/−0.04)
*ECHB*	24.24 ± 15.68	33.45 ± 14.29	0.62	Medium (−1.43/0.22)
*EOCS*	27.19 ± 14.44	40.55 ± 20.89	0.70	Medium (−1.51/0.15)
**COP-AV Percentage Change from *CONTROL***				
*ECHB*	94.03 ± 98.22	45.49 ± 51.52	0.69	Medium (−0.16/1.49)
*EOCS*	132.17 ± 89.39	77.94 ± 85.33	0.63	Medium (−0.22/1.43)

^*^ significant gender difference at p<0.05.

COP-PV=COP path velocity; COP-RD=COP radial displacement; COP-AV=COP area velocity; ECHB=eyes closed, head tilted back at 45°; EOCS=eyes open, compliant (foam) surface; CI=confidence interval

**Table 2:** Correlations between postural stability (pooled conditions) and other variables

	**COP-PV(mm/s)**	**COP-RD (mm/m)**	**COP-AV (mm^2^/s)**	**Average**
**Girls (n = 9)**				
*Age (yrs)*	−0.83 ^*^	−0.41 ^*^	−0.63 ^*^	−0.62
*Body height (m)*	−0.89 ^*^	−0.45 ^*^	−0.68 ^*^	−0.67
*Body mass (kg)*	−0.86 ^*^	−0.33	−0.61 ^*^	−0.60
*BMI (kg/m^2^)*	−0.64 ^*^	−0.16	−0.42 ^*^	−0.41
*Foot Length (m)*	−0.91 ^*^	−0.44 ^*^	−0.70 ^*^	−0.68
*PA Level*	−0.44 ^*^	−0.39 ^*^	−0.51 ^*^	−0.45
**Boys (n = 17)**				
*Age (yrs)*	−0.36 ^*^	−0.37 ^*^	−0.47 ^*^	−0.40
*Body height (m)*	−0.45 ^*^	−0.34 ^*^	−0.43 ^*^	−0.41
*Body mass (kg)*	−0.52 ^*^	−0.32 ^*^	−0.41 ^*^	−0.42
*BMI (kg/m^2^)*	−0.29 ^*^	−0.05	−0.08	−0.14
*Foot Length (m)*	−0.44 ^*^	−0.42 ^*^	−0.49 ^*^	−0.45
*PA Level*	−0.06	0.07	0.05	0.02

^*^ significant correlation at p<0.05.

*COP-PV=COP path velocity; COP-RD=COP radial displacement; COP-AV=COP area velocity; BMI=Body Mass Index; PA=physical activity*.
